# The Association Between Smokeless Tobacco and a Lung Mass in a Healthy Young Male

**DOI:** 10.7759/cureus.36467

**Published:** 2023-03-21

**Authors:** Hossny Alaws, Tanya Aggarwal, Hadia R Ahmad, Chehada A Hatoum

**Affiliations:** 1 Internal Medicine, Northeast Georgia Medical Center Gainesville, Gainesville, USA; 2 Internal Medicine, Northeast Georgia Medical Center Gainesville, Braselton, USA

**Keywords:** chewing tobacco, mass lesion lung, smokeless tobacco, streptococcus intermedius, lung abscess

## Abstract

Smokeless tobacco is widely used in the United States. Many commonly used forms of smokeless tobacco may contain microorganisms that can change the oral flora of tobacco users. Here we present a case of a previously healthy 21-year-old male who presented with six weeks of worsening cough productive of yellow sputum as well as pleuritic left-sided chest and back pain. Computed tomography (CT) of the chest showed a large 3.9 x 5.5 x 6.3 cm mass-like lesion.

He was extensively worked up for potential causes of this mass, including autoimmune, HIV testing, sputum staining for acid-fast bacilli, and fungal serologies. He was empirically treated with antibiotics and antifungals. He ultimately underwent a CT-guided biopsy which was negative for malignancy and tuberculosis. The culture from the biopsy revealed 5,000 colony forming units of Streptococcus intermedius. Based on the sensitivities of the culture, he was switched to intravenous ceftriaxone and discharged to complete a course of intravenous antibiotics.

This case showcases a healthy 21-year-old male with no prior history who had an extensive workup for the possible causes and risk factors predisposing to a lung abscess. This workup was negative, and his only risk factor was the use of smokeless chewing tobacco. Smokeless tobacco may be associated with increased risk of lower respiratory tract infections and can increase the risk of lung abscess in an immunocompetent adult. More research is required to understand this association.

## Introduction

Smokeless tobacco is widely used in the United States, with over 3.6% of adults above the age of 18 using some form of smokeless tobacco [1-2]. There are multiple forms of smokeless tobacco [2]. Some studies suggest there is a higher prevalence of bacterial species that increase inflammation in the oral microbiome in people who consume smokeless tobacco products [3]. One study looked at the effects of smokeless tobacco on the oral microbiome of healthy adults and those with oral squamous cell carcinoma and found a high prevalence of Streptococcus, Prevotella, and Fusobacterium in the oral flora of smokeless tobacco users [3]. Another study showed 493 genera of microorganisms in smokeless tobacco products, the most abundant of which was Staphylococcus, followed by Bacillus and Corynebacterium, Prevotella, Pantoea, Streptococcus, Leptotricia, Propionibacterium, Rothia, Fusobacterium, Veillonella and Weisella [4]. When smokeless tobacco, especially in form of dip, is kept in the gingivobuccal sulcus, there is a high risk of chronic microaspiration into the lungs causing recurrent pneumonia and abscess formation [5].

During swallowing, the epiglottis retroflexes posteriorly, closing the larynx, which protects the airways from aspiration of food content [6]. Alterations in the epiglottis retroflection mechanism can increase risk of aspiration up to four times [6-7]. 

Here, we present a case of a previously healthy 21-year-old male who presented with a large lung mass which was extensively investigated and was found to be an abscess secondary to Streptococcus intermedius. The patient reported some aspiration symptoms and is a chronic user of chewing tobacco. The remainder of the workup was unremarkable, suggesting that smokeless tobacco use was the most likely risk factor for developing this abscess. 

## Case presentation

A 21-year-old male with no known medical history presented to the emergency department with six weeks of worsening cough productive of yellow sputum as well as pleuritic left-sided chest and back pain and night sweats. He has no known family history of genetic conditions. Social history was significant for smokeless tobacco use in the form of chewing tobacco.

**Figure 1:**
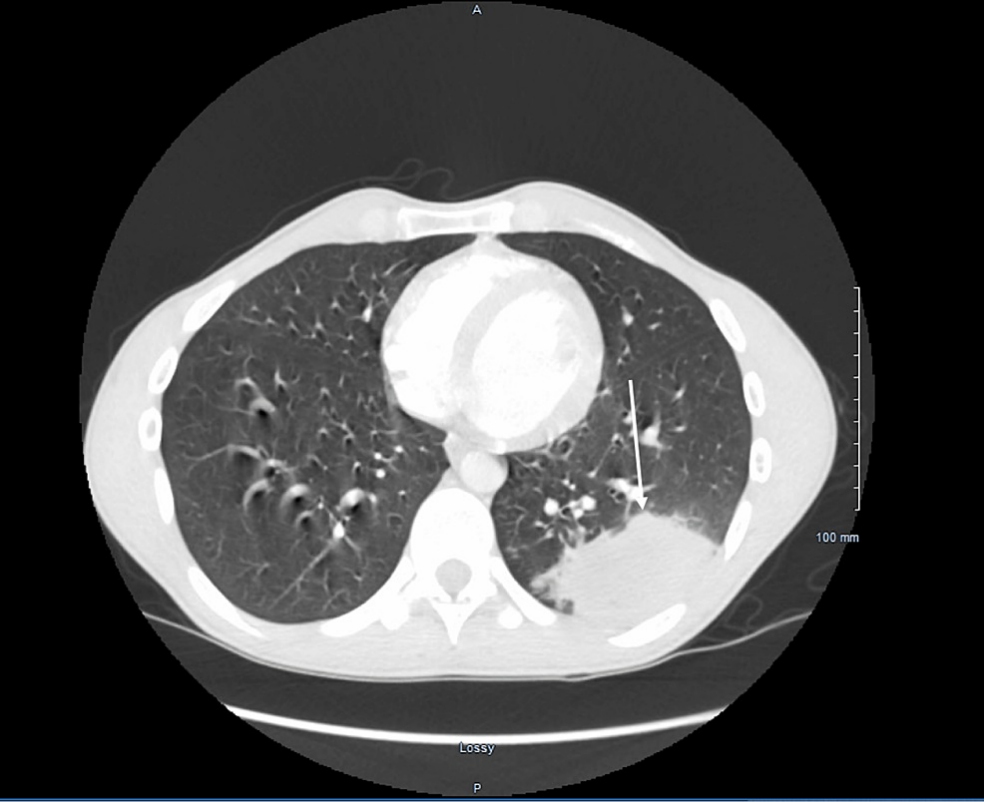
Transverse section of a Computed Tomography showing a 3.9 x 5.5 x 6.3 cm mass-like consolidative change in the left lung

**Figure 2:**
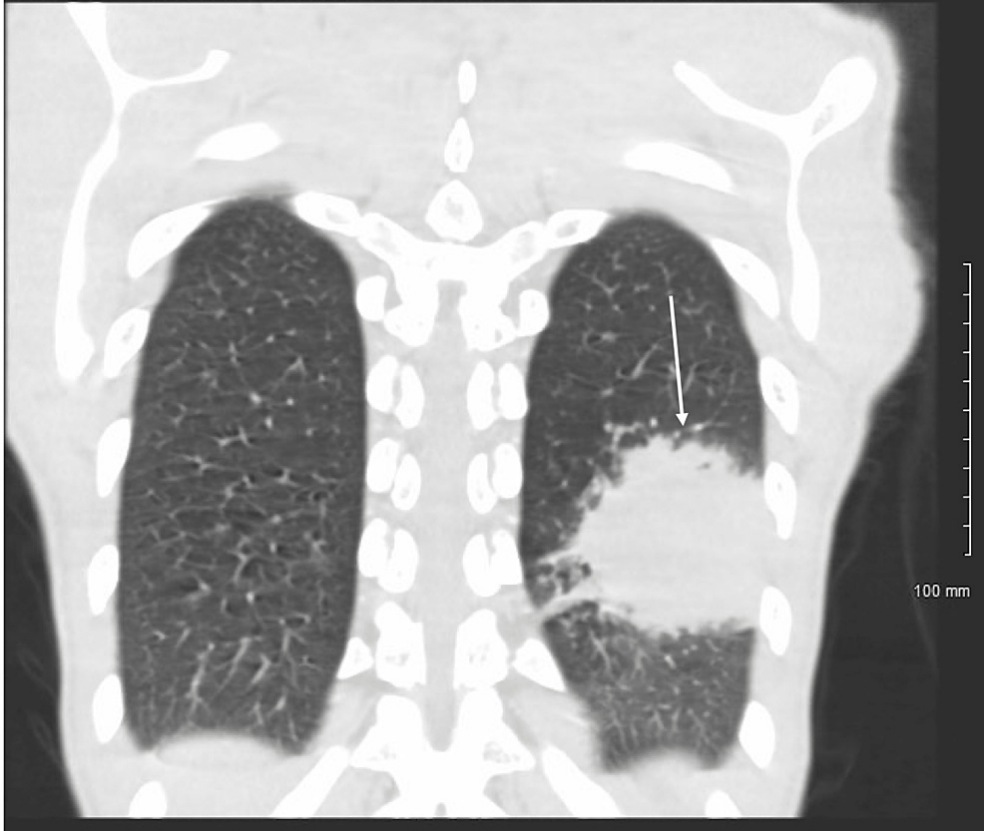
Coronal section of a computed tomography slice showing the lung mass in the left lobe

On admission, he had an elevated white cell count of 12,200. Computed tomography of the chest showed a large 3.9 x 5.5 x 6.3 cm mass-like lesion (Figures 1, 2). He was extensively worked up for potential causes of this mass. HIV testing was negative. Fungal causes were investigated with sputum potassium hydroxide preparation, Cryptococcal serum antigen, Histoplasma, and Blastomyces urine antigen, which were negative. Cluster of differentiation (CD) 4 and CD8 counts were within normal limits. Blood and sputum bacterial and fungal cultures were negative. Autoimmune testing, Quantiferon testing as well as sputum staining for acid-fast bacilli were negative. 1,3-β-D-glucan and galactomannan were also negative.

The patient was empirically started on piperacillin/tazobactam as well as itraconazole on admission. He subsequently had a flexible bronchoscopy with bronchoalveolar lavage, bronchial brushing, and endobronchial biopsy, which were negative for malignancy and tuberculosis. Notably, a barium swallow was done due to a history of coughing and choking with swallowing; this revealed an abnormally elongated epiglottis with incomplete inversion during swallowing.

**Figure 3:**
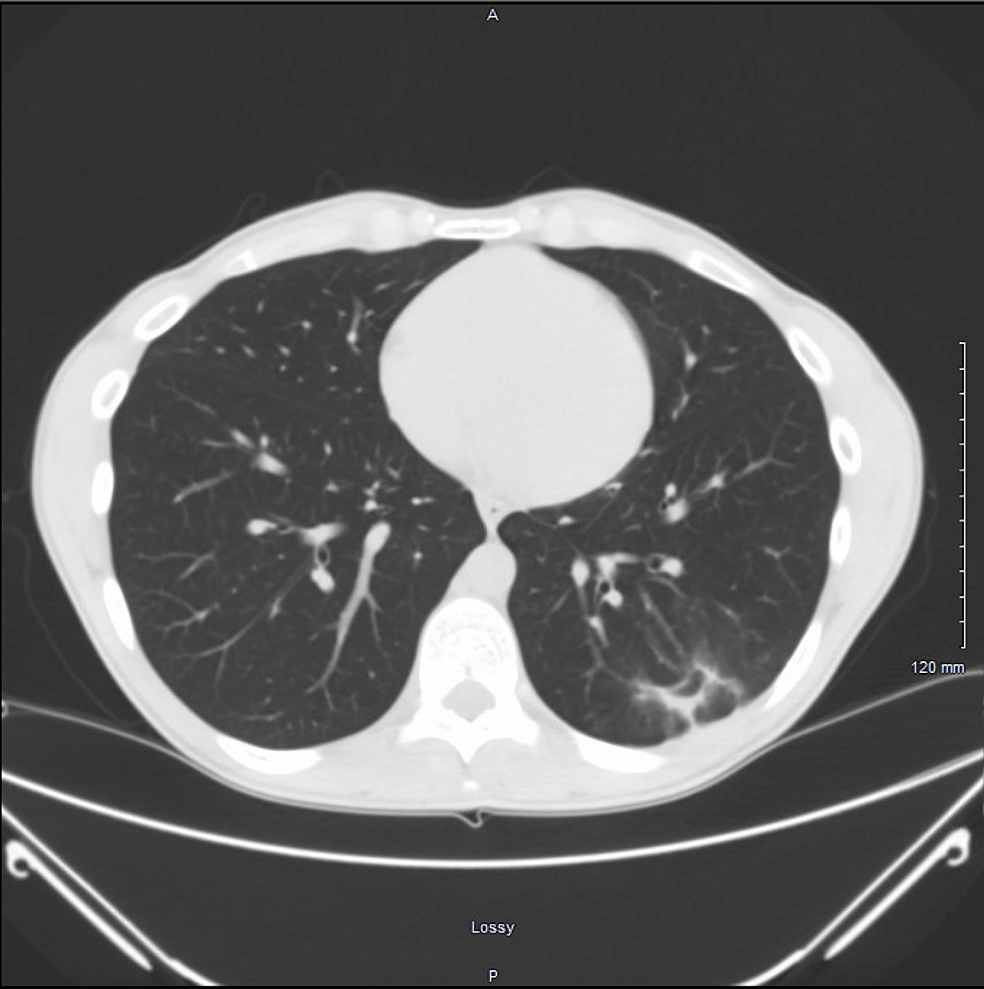
Transverse computed tomography image showing resolution of the lung mass with residual cavitation in its place

**Figure 4:**
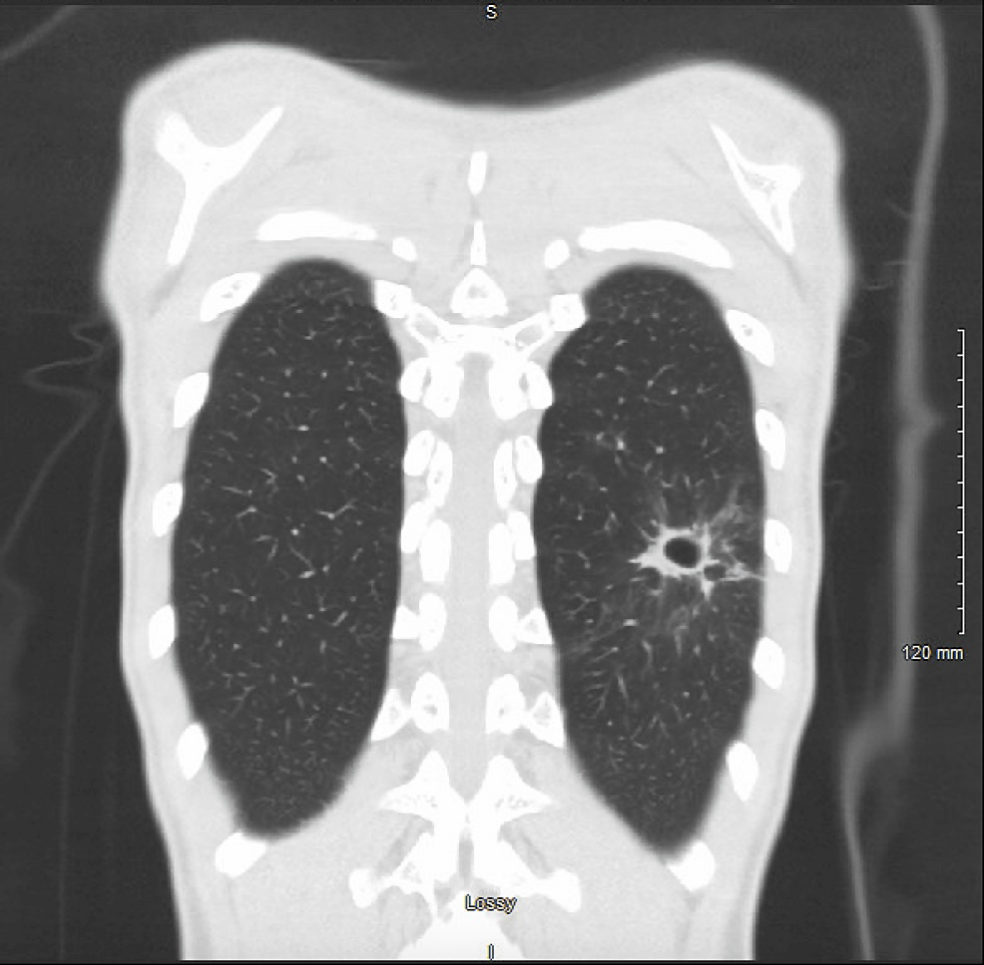
Coronal section of a computed tomography image showing resolution of the lung mass with residual cavitation in its place

Days following his admission, one of the cultures from the bronchoalveolar lavage yielded 5,000 colony forming units of Streptococcus intermedius. A computed-tomography-guided biopsy done after the bronchoscopy showed organizing pneumonia with focal necrosis. He was switched to intravenous ceftriaxone based on sensitivities and discharged to complete a course of intravenous antibiotics. A follow-up computed tomography of the chest without contrast showed resolution of the mass with focal cavitation in its place following the completion of antibiotics (Figures 3, 4). The patient has done well since, with no respiratory symptoms.

## Discussion

This patient presented with a large lung mass and underwent a very extensive workup for possible causes, including tuberculosis, common fungal infections, autoimmune, leukemia, lymphoma, and malignancy. This workup was unremarkable. The only revealing finding on this patient’s workup was a positive bronchoalveolar lavage culture for Streptococcus intermedius, indicating an infectious etiology to this mass.

The patient does not have any family history of lung conditions or autoimmune conditions. He is not immunocompromised, and HIV testing was negative. Given these findings, the only possible explanation for developing a lung abscess in an otherwise healthy, immunocompetent patient is his use of smokeless tobacco combined with aspiration in the setting of an elongated epiglottis with inadequate retroflection. An elongated epiglottis represents a significant risk factor for aspiration, as one study demonstrates that 56% of patients with alterations in epiglottic movement aspirate compared to 18% who did not [7]. Epiglottic retroflection is critical for airway protection during swallowing [8]. Reduced laryngeal elevation and reduced tongue base retraction underlie impaired epiglottic retroflection. Dysfunction of epiglottic retroflection may be due to direct trauma to the fibroelastic body, the prolonged presence of a tube displacing the epiglottis, or some other destructive disease process (such as chondromalacia, tumor formation, arteriovenous malformation) but none of these were present in our patient [8].

Evidence in the literature is lacking regarding the extent of the association between smokeless tobacco use and lung infections or abscesses.

## Conclusions

This case highlights an association between smokeless tobacco and lung infections in an otherwise immunocompetent young adult. More research is needed to determine the extent of the association between smokeless tobacco use and lung infections.
